# *Streptococcus suis* Meningitis without History of Animal Contact, Italy

**DOI:** 10.3201/eid1412.080679

**Published:** 2008-12

**Authors:** Aldo Manzin, Claudio Palmieri, Corrado Serra, Barbara Saddi, Maria Stella Princivalli, Giovanni Loi, Giuseppe Angioni, Franco Tiddia, Pietro E. Varaldo, Bruna Facinelli

**Affiliations:** University of Cagliari Medical School, Cagliari, Italy (A. Manzin, C. Serra); Polytechnic University of Marche Medical School, Ancona, Italy (C. Palmieri, M.S. Princivalli, P.E. Varaldo, B. Facinelli); SS. Trinità Hospital, Cagliari (B. Saddi, G. Angioni, F. Tiddia); Azienda Ospedaliero-Universitaria, Cagliari (G. Loi)

**Keywords:** Streptococcus suis, bacterial meningitis, human, pigs and other animals, letter

**To the Editor:** *Streptococcus suis*, a major swine pathogen worldwide, is emerging as a zoonotic agent capable of causing a variety of serious infections in swine as well as in persons exposed to pigs or to pork products. These infections include meningitis, septicemia, pneumonia, endocarditis, arthritis, and septic shock (*1*,*2*). Despite recent outbreaks among persons in China, *S. suis* disease in humans is a rare, probably underdiagnosed infection that usually occurs as sporadic cases (*1*,*2*). Persons in close occupational or accidental contact with pigs or pork products and those who eat uncooked or undercooked pork may be at higher risk than others. However, most infected persons are likely healthy carriers, and *S. suis* is believed to induce overt disease (especially meningitis) in only some circumstances (*2*). We describe a case of *S. suis* meningitis in a 68-year-old man from Sardinia, Italy, who had no reported contact with swine, other animals, or any animal products; the patient also had cancer, which was discovered incidentally during the workup.

In November 2007, the patient was hospitalized with a 48-hour history of fever, headache, nausea, and general malaise. Physical examination showed impaired consciousness, nuchal rigidity, and a temperature of 39.5°C. Laboratory findings were 20,700 leukocytes/mm^3^ with 92% neutrophils, glucose 95 mg/dL, and C-reactive protein 375 mg/L. Cerebrospinal fluid (CSF) analysis demonstrated 240 leukocytes/μL with 80% polymorphonuclear cells, glucose 24 mg/dL, and protein 277 mg/dL. A computed tomography scan of the head showed no abnormal findings. Gram stain of CSF showed gram-positive cocci, mostly in pairs (Figure).

**Figure:**
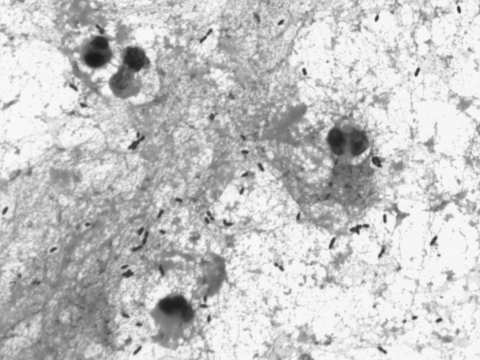
Gram-positive cocci, mostly in pairs, in cerebrospinal fluid from a 68-year-old man with *Streptococcus suis* meningitis. Magnification ×1,000.

Empirical therapy consisted of intravenous ceftriaxone (2 g twice a day) and oral chloramphenicol (2 g once a day). On day 5, α-hemolytic streptococci were isolated from CSF on sheep blood agar and identified as *S. suis* by using APIStrep (bioMérieux, Marcy l’Etoile, France). Serotyping, performed by slide agglutination with specific antiserum (Statens Serum Institute, Copenhagen, Denmark), identified the isolate as serotype 2.

Antimicrobial drug­­–susceptibility testing, performed according to guidelines of the Clinical and Laboratory Standards Institute (www.clsi.org), indicated susceptibility to penicillin, ceftriaxone, chloramphenicol, levofloxacin, and vancomycin and resistance to erythromycin (MIC >128 mg/L) and tetracycline (MIC 16 mg/L). Erythromycin resistance was constitutive and was mediated by the *erm*(B) determinant; tetracycline resistance was mediated by *tet*(W). Multilocus sequence typing (http://ssuis.mlst.net) assigned the *S. suis* isolate to sequence type (ST) 1.

The patient, a retired welder, denied any recent occupational or even occasional contact with swine or other animals and had no history of eating raw or undercooked pork. The patient’s condition improved; chloramphenicol was discontinued on day 10, but the 14-day course of ceftriaxone was completed. On day 6, the patient became afebrile but had dizziness and deafness; a formal audiology evaluation on day 9 showed severe bilateral sensorineural high-frequency hearing loss (–80 dB) that improved after a short course of dexamethasone. However, the patient was not discharged because of the lung mass found on initial chest radiograph. Computed tomography scan, bronchoscopy, and histopathologic findings led to diagnosis of the mass as an advanced-stage squamous cell carcinoma.

The meningitis had common and uncommon features. The common features were hearing loss, a typical outcome of *S. suis* meningitis independent of early antimicrobial drug administration (*1*,*2*); serotype 2, the most frequent and virulent serotype in swine and in humans (*1*,*2*); ST1, belonging to the ST1 complex, strongly associated with *S. suis* meningitis isolates (*2*,*3*); and *erm*(B)-mediated erythromycin resistance, widespread in this species (*4*). The uncommon features were tetracycline resistance mediated by *tet*(W), increasingly detected in gram-positive and in gram-negative bacteria (*5*) but never previously reported in *S. suis* or in other major streptococcal pathogens, where common determinants are *tet*(M) and *tet*(O); and lack of evidence for recent contact with swine, other animals, or swine (pork) products.

Two previous cases of human *S. suis* meningitis in Italy (*6*,*7*) and other recent cases from Europe (*8*,*9*) were related to occupational exposure. However, the patient reported here also had cancer, and malignancy has been indicated as a predisposing factor for the development of severe *S. suis* disease in humans (*2*). These findings appear to be consistent with the recent suggestion of new epidemiologic patterns of infection caused by this organism (*2*). *S. suis* may become an opportunistic pathogen in persons who are under stress or who have immunodeficiency, and it has been increasingly isolated from mammalian species other than pigs, from birds, and from the environment. As also discussed in a recent survey (*10*), the possibility cannot be excluded that a patient with *S. suis* infection may be unaware or have no memory of previous exposure to animals. Alternatively, because asymptomatic carriage of *S. suis* has been documented in humans (*2*) and is believed to contribute to its transmission (*10*), the possibility should also be considered that the infection may be a reactivation, possibly favored by malignancy, of latently colonizing *S. suis*.
